# ﻿Two new snake eels (Anguilliformes, Ophichthidae, *Ophichthus*) from Viet Nam, with redescriptions of *O.macrochir* (Bleeker) and *O.rutidoderma* (Bleeker)

**DOI:** 10.3897/zookeys.1231.137323

**Published:** 2025-03-13

**Authors:** Quang Van Vo, Yusuke Hibino, Hsuan-Ching Ho, Thao Thu Thi Le, Ying Giat Seah

**Affiliations:** 1 Institute of Oceanography, Vietnam Academy of Science and Technology, No. 01 Cau Da, Nha Trang City, Khanh Hoa, Vietnam Institute of Oceanography, Vietnam Academy of Science and Technology Nha Trang Vietnam; 2 Kitakyushu Museum of Natural History and Human History, Kitakyushu, Fukuoka, Japan Kitakyushu Museum of Natural History and Human History Kitakyushu Japan; 3 Graduate Institute and Department of Aquaculture, National Kaohsiung University of Science and Technology, Kaohsiung, Taiwan National Kaohsiung University of Science and Technology Kaohsiung Taiwan; 4 Australia Museum, Sydney, Australia Australia Museum Sydney Australia; 5 Faculty of Fisheries and Food Science, Universiti Malaysia Terengganu, Kuala Nerus, Terengganu 21030, Malaysia Universiti Malaysia Terengganu Kuala Nerus Malaysia

**Keywords:** Biodiversity, Elopomorpha, ichthyology, Mekong, Ophichthinae, *Ophichthuscuulongensis* sp. nov., *Ophichthusnguyenorum* sp. nov., taxonomy

## Abstract

Two new extremely elongate snake eel of the genus *Ophichthus* are described based on specimens collected from Vietnamese waters. *Ophichthuscuulongensis* Vo, Hibino & Ho, **sp. nov.** is distinguished from its congeners by having the dorsal-fin origin slightly behind the pectoral-fin tip, mean vertebral formula 14-63-202, range 12–17/60–64/199–207; teeth on jaws biserial to triserial; dorsal body dark brown, ventral body pale, anal fin initially white but changing to darker towards its tip. *Ophichthusnguyenorum* Vo, Hibino & Ho, **sp. nov.** is distinguished by having a snout rather pointed but the occipital convex (duck-shaped); body with numerous longitudinal wrinkles, weak on posterior abdomen; dorsal-fin origin slightly behind the pectoral-fin tip; one row of teeth on the maxilla anteriorly but increasing posteriorly; two rows on the lower jaw; all teeth small; body dark, usually including abdomen; dorsal fin darker with dark margin; anal fin initially pale but changing to darker towards tip; mean vertebral formula: 15-62-192, range 13–17/61–64/190–196. Descriptions of two related species, *O.macrochir* (Bleeker, 1852) and *O.rutidoderma* (Bleeker, 1852), are provided with updated morphological data.

## ﻿Introduction

The family Ophichthidae has the highest number of species among the anguilliform families, including more than 300 species distributed among 62 genera (Fricke et al. 2024). Many new species belonging to the snake eel genus *Ophichthus* have been described in recent decades (McCosker 2010; McCosker et al. 2012; McCosker and Ho 2015; McCosker and Psomadakis 2018; Hibino et al. 2019a, b; McCosker et al. 2020; Vo and Ho 2021; Ho et al. 2022; Kodeeswaran et al. 2024a, b; Behera et al. 2024; Hibino et al. 2024). The genus *Ophichthus* (Ahl, 1789) is a diverse genus of the family Ophichthidae with more than 100 valid species (Fricke et al. 2024). The genus *Ophichthus* can be separated into two groups based on body shape: one group with the body stout to moderately elongate, its depth behind gill openings < 40 in TL and the second group with body shape elongate to extremely elongate, its depth behind gill openings > 40 in TL (McCosker et al. 2020).

The genus *Ophichthus* comprises 17 species in Vietnam with some new species and records reported recently (Vo et al. 2024). Historically, only three species with elongated to extremely elongated bodies, *Ophichthusmacrochir* (Bleeker, 1852), *O.microcephalus* Day, 1878, and *O.rutidoderma* (Bleeker, 1852), were found in Viet Nam (Nguyen 1993; Nguyen and Nguyen 1994; Nguyen 2001; Vo et al. 2024). However, *O.microcephalus* is a questionable record due to the lack of firm evidence. In the Mekong estuary region, several elongate snake eels were identified as *O.rutidoderma* (and its synonym) or *Pisodonophisboro* (Hamilton, 1822) by previous authors (Mai et al. 1992; Rainboth 1996; Rainboth et al. 2012; Tran et al. 2013; Nagao Natural Environment Foundation 2021). However, we could not confirm those records based on only the brief information or photographs.

Recently (2023–2024), surveys of the family Ophichthidae in the Mekong estuary water were conducted and many specimens were collected. The morphological characteristics revealed that two new species in the genus *Ophichthus* are distinct from its congeners. Moreover, two other extremely elongated congeners, *O.macrochir* and *O.rutidoderma*, both lacking sufficient morphological data, are redescribed based on the types and additional specimens collected from various localities.

## ﻿Materials and methods

All methods for counts and measurements follow McCosker (2010), with further explanation below. Measurements for total and tail lengths are taken by 600- or 1000-mm rulers and others by digital caliper to the nearest 0.1 mm. Vertebral counts were made from radiograph films or digital radiograph photographs follow Böhlke (1982). Mean vertebral formula (**MVF**) is expressed as the average of predorsal, preanal and total vertebrae and vertebral formula (**VF**) are the solo number of each. The vertebral count includes the hypural. Terminology of head structures around lips and head pore system follow Hibino et al. (2019a, b), and are abbreviated as **SO** (supraorbital pores), **IO** (infraorbital pores), **POM** (preoperculomandibular pores), and **ST** (supratemporal pores). Lateral-line pores: head pores (**HLL**), pores before dorsal-fin origin (**PDLL**), pores before anal-fin origin (**PALL**) and total pores (**TLL**). Dorsal-fin origin (**DFO**) and anal-fin origin (**AFO**) are abbreviated. Total and head lengths are abbreviated as **TL** and **HL**, respectively.

Alcian blue was used to stain the skins of most specimens in order to make the precise counts of pores. Radiographs were made by a digital x-ray machine set up at the National Museum of Marine Biology & Aquarium, Taiwan, with pins inserted at origins of dorsal and anal fins. Specimens were deposited at the Natural History Museum, London, UK (**BMNH**); Pisces collection of National Museum of Marine Biology & Aquarium, Pingtung, Taiwan (**NMMB-P**); Kagoshima University Museum, Kagoshima, Japan (**KAUM-I**); Kitakyushu Museum of Natural History and Human History, Kitakyushu, Fukuoka, Japan (**KMNH VR**); and Institute of Oceanography, Nha Trang, Vietnam (**OIM-E**), Vietnam. Data used for comparison were either taken from specimens examined by the authors and various publications as indicated.

## ﻿Taxonomic accout

### ﻿Family Ophichthidae

**Genus *Ophichthus* Ahl, 1789**

#### ﻿ Ophichthus cuulongensis

Taxon classificationAnimaliaAnguilliformesOphichthidae

Vo, Hibino & Ho sp. nov.

DC70DB39-3607-5531-AE20-1EA8394A0CDB

https://zoobank.org/8E3B8CBF-A165-4153-89CA-D656639B159A

**Figure 1.**
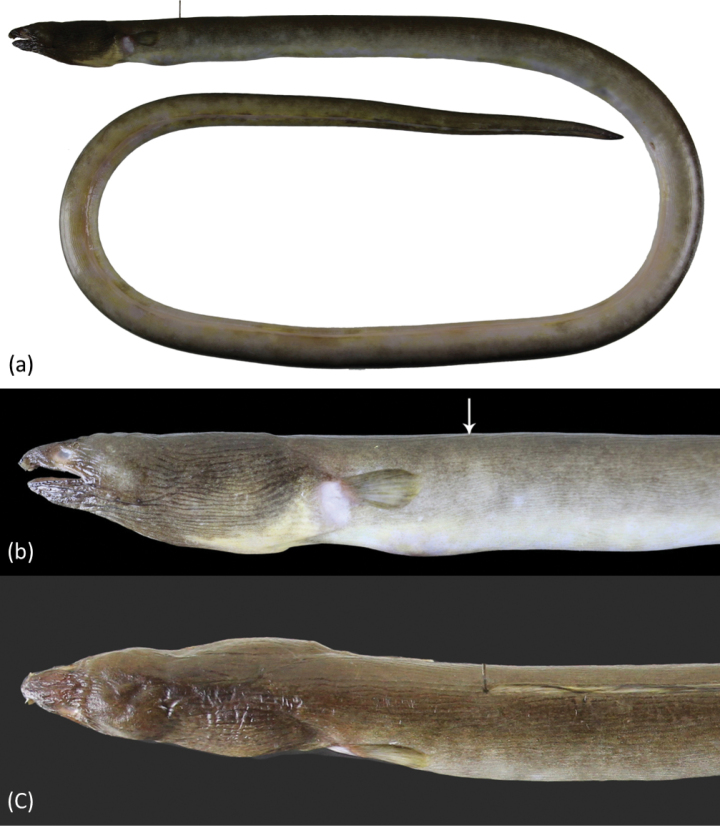
*Ophichthuscuulongensis* sp. nov., holotype, OIM-E. 55819, 904 mm TL**a** fresh specimen, arrows point to the DFO and AFO, respectively **b** close-up of head view from lateral side, arrow points to the DFO**c** close-up of head view from dorsal.

**Figure 2.**
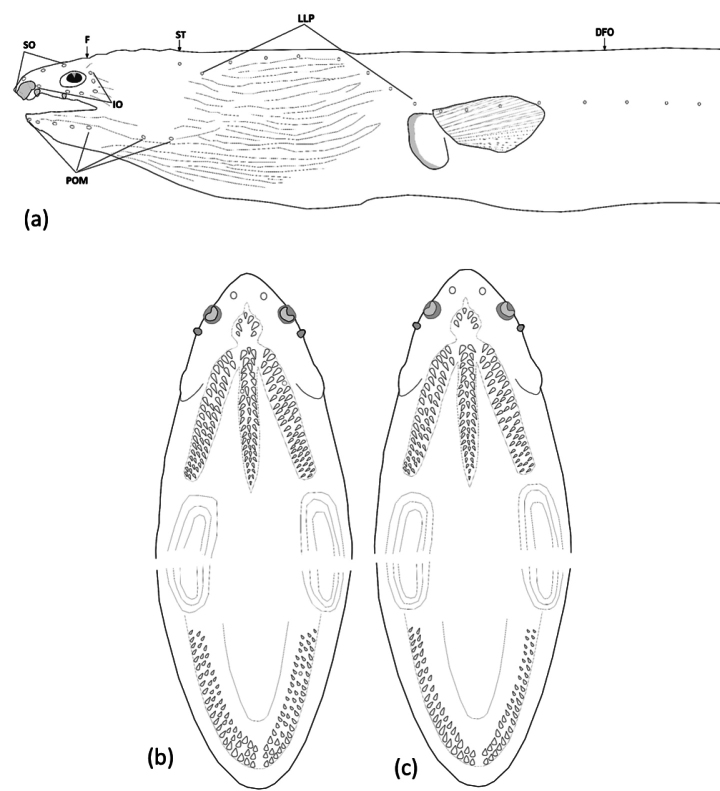
Drawing demonstrating the head pores (**a**) and teeth on both jaw (**b, c**) of *Ophichthuscuulongensis* sp. nov. **a, b** from the holotype **c** from OIM-E.55824, paratype, 808 mm TL.

Figs 1 , 2 , Tables 1 , 2 English name: Mekong’s Estuary Snake Eel Vietnamese name: Cá lịch mỡ

##### Type material.

***Holotype***: • OIM-E. 55819, 904 mm TL, ripe female, field no. Q.01020, ca 12°19'N, 109°20'E, Đồng Hòa, Cần Giờ, Hồ Chi Minh city, southeast coast of Vietnam, South China Sea, bottom trawl, ca 10–20 m, 28 Aug. 2023. ***Paratypes***: • Fifty seven specimens, 475–998 mm TL, all collected from some sites, including Đồng Hòa port fishing (10°22'57.45"N, 106°53'0.91"E), Cần Giờ district, Hồ Chí Minh city and Tân Bình market (ca 10°0'14.77"N, 106°37'23.73"E) and the old Ba Tri market (ca 10°2'25.96"N, 106°35'38.87"E), Ba Tri district and the Khâu Băng market (ca 9°49'33.35"N, 106°36'3.37"E), Thạnh Phú district, Bến Tre province: NMMB-P41234, 14 specimens, 640–831 mm TL, collected in 2020, 2023 & 2024 • OIM-E.55812, 945 mm TL, 13 Nov. 2013 • OIM-E.55813, 475 mm TL, 16 Sep. 2014 • OIM-E.55814, 783 mm TL, 13 Nov. 2014 • OIM-E.55815, 586 mm TL, 16 Sep. 2014 • OIM-E.55816, 880 mm TL, 10 Sep. 2016 • OIM-E.55817, 7 specimens, 640–872 mm TL, 06 & 08 Sep. 2020 • OIM-E.55818, 2 specimens, 710–725 mm TL, 22 Jun. 2023 • OIM-E.55820, 2 specimens, 696–781 mm TL, 20 Sep. 2023 • OIM-E.55821, 8 specimens, 732–878 mm TL, 23 Sep. 2023 • OIM-E.55822, 673 mm TL, 12 Oct. 2023 • OIM-E.55823, 2 specimens, 640–688 mm TL, 15 Oct. 2023 • OIM-E.55824, 10 specimens, 544–994 mm TL, 10 & 12 Nov. 2023 • OIM-E.55825, 2 specimens, 784–810 mm TL, 19 Jan. 2024. KMNH VR 100622, 4 specimens, 625–830 mm TL, collected in 2020 & 2023.

**Table 1.** Morphometric and meristic data of four elongate *Ophichthus* species.

	*O.cuulongensis* sp. nov.			*O.nguyenorum* sp. nov.			*O.macrochir*		*O.rutidoderma*	
	Holotype	All types		Holotype	All types		Non-types		All types	
Total length (mm)	904	547–998 (*n* = 58)		887	697–967 (*n* = 18)		324–823 (*n* = *8*)		415–867(*n* = 11)	
**Proportions**		Mean (Range)	SD		Mean (Range)	SD	Mean (Range, n)	SD	Mean (Range, n)	SD
As % of TL										
Head length	5.8	5.7 (5.2–6.2)	0.3	5.4	5.7 (5.4–6.2)	0.3	5.9 (5.6–6.7, 7)	0.4	6.3 (5.7–7.2, 8)	1.1
Preanal length	33.1	32.7 (31.3–34.6)	0.8	32.0	32.8 (31.4–33.5)	0.6	35.5 (31.0–37.4, 7)	1.6	33.1 (32.2–34.1, 8)	1.3
Trunk length	27.2	27.0 (25.3–28.5)	0.8	26.6	27.1 (25.9–28.0)	0.5	29.6 (25.2–31.7, 7)	1.6	26.8 (26.2–27.8, 8)	1.2
Tail length	66.9	67.3 (65.4–68.8)	0.8	68.0	67.2 (66.5–68.6)	0.6	64.5 (62.5–69.3, 7)	1.7	66.8 (65.7–67.5, 8)	1.2
Predorsal length	8.1	8.2 (7.1–9.5)	0.5	7.6	8.3 (7.6–8.7)	0.4	7.3 (6.7–7.9, 7)	0.4	8.7 (8.0–9.4, 8)	0.9
Body depth at gill opening	1.8	1.6 (1.5–1.8)	0.1	1.6	1.6 (1.6–1.7)	0.1	1.6 (1.5–1.8, 6)	0.1	1.9 (1.5–2.1, 8)	0.4
Body width at gill opening	1.4	1.5 (1.4–1.7)	0.1	1.5	1.5 (1.5–1.6)	0.0	1.4 (1.4–1.5, 3)	0.1	1.8 (1.6–2.3, 6)	0.5
Body depth at mid-anus	1.8	1.8 (1.6–2.0)	0.1	1.5	1.5 (1.5–1.6)	0.1	1.6 (1.3–1.8, 6)	0.2	2.2 (1.8–2.6, 8)	0.5
Body width at mid-anus	1.8	1.8 (1.6–2.0)	0.1	1.4	1.4 (1.4–1.5)	0.0	1.6 (1.5–1.9, 3)	0.3	2.2 (1.9–2.5, 6)	0.5
As % of HL										
Snout length	13.7	14.3 (12.9–15.3)	0.7	15.3	15.7 (14.6–16.4)	0.5	17.0 (13.8–19.8, 7)	1.7	15.0 (12.4–16.9, 8)	3.2
Eye diameter	6.8	6.8 (6.3–7.3)	0.3	5.9	6.2 (5.8–6.6)	0.2	7.1 (5.1–9.3, 7)	1.4	5.7 (4.0–7.1, 8)	2.2
Upper-jaw length	25.8	25.1 (23.3–26.4)	1.0	29.5	29.6 (28.1–31.1)	0.8	33.0 (28.1–41.9, 7)	3.4	27.4 (22.6–29.7, 8)	5.0
Low-jaw length	22.6	21.6 (19.0–23.1)	0.9	24.7	25.3 (24.7–26.3)	0.6	24.4 (24.2–24.9, 6)	0.9	24.2 (22.5–25.8, 2)	2.3
Gill-opening length	13.1	13.9 (12.5–15.9)	0.9	17.6	17.3 (16.5–18.0)	0.5	11.5 (7.4–17.4, 7)	4.6	15.1 (8.1–20.2, 8)	8.6
Interorbital width	12.7	13.3 (12.1–14.3)	0.6	13.00	13.2 (12.5–14.0)	0.4	13.2 (9.9–15.9, 7)	1.5	13.4 (11.8–16.1, 8)	3.1
Isthmus width	19.4	17.9 (15.8–19.9)	0.9	15.3	15.0 (14.1–16.0)	0.6	18.5 (16.6–20.7, 6)	1.8	16.8 (14.6–19.0, 2)	3.1
Pectoral-fin length	25.8	29.2 (22.9–34.1)	2.5	31.2	31.0 (28.4–33.6)	1.7	29.3 (27.0–32.9, 7)	4.6	30.3 (28.1–32.9, 7)	3.4
**Counts**	–	*n* = 46	–	–	*n* = 18	–	*n* = 3	–	*n* = 9	–
PALL	62	63 (62–65)	–	62	62 (61–64)	–	71 (70–73)	–	63 (59–67)	–
Predorsal vertebrae	15	14 (12–17)	–	15	15 (13–17)	–	11 (11–12)	–	16 (14–16)	–
Preanal vertebrae	63	62 (60–64)	–	63	62 (61–64)	–	70 (69–71)	–	63 (60–68)	–
Total vertebrae	201	202 (199–207)	–	192	192 (190–196)	–	217(214–221)	–	195 (191–199)	–

##### Diagnosis.

An extremely elongate snake eel species of the genus *Ophichthus* with the following combination of characters: occipital not convex prominently, dorsal of snout with median shallow groove, reaching to interorbital pore; three or more shallow wrinkles (usually 3) on posterior part of eye; body with numerous longitudinal wrinkles, also prominent on abdomen; head length 5.2–6.2% TL; tail length 65.4–68.8% TL; two protrusions along upper lip from each side (some paratypes 1 on one side); dorsal-fin origin slightly behind pectoral-fin tip; SO 1 + 3, POM 5 (or rarely 6) + 2; teeth small (but larger in intermaxillary and anterior vomer); body dark brown, abdomen generally paler; dorsal fin with dark margin entirely, anal fin initially pale but in posterior part with faded dark margin, the area more than two head length; total vertebrae 199–207, MVF 14-62-202.

##### Description.

Counts and measurements of the holotype (in mm). Total length 904, head 52.7, trunk 246.3, tail 605, predorsal length 73.1, pectoral-fin length 13.6; body depth at gill opening 16.2; body width at gill opening 13.0; body depth at anus 16.5; body width at anus 16.3; snout 7.2; upper jaw 13.6; snout overhang beyond tip of lower jaw 3.0; eye diameter 3.6; interorbital width 6.7; gill opening height 6.9; isthmus width 10.2.

Body extremely elongate (Fig. 1a), subcircular to posterior portion of tail, then becoming slightly compressed, its depth at gill openings 62 (55–69) in TL. Branchial basket slightly expanded and deeper than trunk. Skin slightly wrinkled behind eye, nape, and body with numerous longitudinal wrinkles, also prominent on abdomen. Head short 17.6 (16.1–19.2) in TL and tail elongate 1.5 in TL. Snout short 7.0 (6.4–8.0), tip broadly conical at dorsal view, and bisected by a median shallow groove; lower jaw included, its tip extending slightly beyond posterior margin of anterior nostril tube; its length 4.6 (4.3–5.3) in HL. Upper jaw moderately long, rictus well behind posterior margin of eye; its length 4.0 (3.8–4.3) in HL.

Eye moderate, at mid of upper jaw, its diameter 3.2 (2.8–3.8) in upper-jaw length and 14.8 (13.1–16.8) in HL. Anterior nostril tubular, extending ventrolaterally from snout, reaching below upper lip and chin when directed downward. Posterior nostril a hole above upper lip, covered by a large flap that extends well below edge of mouth gape. Two barbels on upper lip (rarely 1 on one side). Dorsal-fin origin behind head, less than one pectoral-fin length behind fin tip and 1.4 (1.2–1.6) HL behind head. Median fins low but obvious, ending approximately one snout length before broadly pointed tail tip. Pectoral fin with narrow base, its length less than three times its base width, broad at middle, the longest rays at mid-fin.

Head pores small but apparent (Fig. 2a). SO 1 (ethmoid) + 3 on dorsal surface of snout and interorbital space; IO 3 + 3, one between nostrils, two below eye, and three behind eye; M 5 (1 paratype with 6 on left side), the last pore slightly before rictus; POP 2, F 1, ST 3. Indistinct minute sensory papillae present along nape, anterior margin of orbit, and around base of anterior nostril. Lateral-line pores apparent; HLL 9 (8–10), in an arching sequence, PDLL 15 (13–17; PALL 63 (62–65); TLL 198 (195–202), the last at approximately an upper-jaw length in front of tail tip.

Teeth (Figs 2b, c) moderately large, conical, and variable sizes. Intermaxillary with 4–6 teeth large and robust, arranged in two rows (some paratypes with a one at center), followed by 19 (17–20) teeth on vomer, biserial rows (some paratypes with 3 irregular rows and uniserial posteriorly), which decrease slightly in size posteriorly. Maxilla with 18–21 (right side) or 17–22 (left side) teeth mostly triserial (some paratypes biserial at anterior portion). Mandible with 17–24 teeth on right side or 18–25 teeth on left side; teeth arranged in two or three well-separated rows anteriorly, gradually becoming two rows at middle then three rows posteriorly, outer row slightly larger and more robust than the inner row (Fig. 2c).

##### Coloration.

When fresh (Fig. 1a) body dark brown; pale white ventrally; dorsal fin dark margin; anal fin initially pale but in posterior part with faded dark margin, the area longer than two times head length; tail tip darker; pectoral fin pale to light yellow. After preservation, body uniformly dark brown dorsally and pale brown ventrally. Snout relatively darker than other skin; branchial basket and chest darker. Dorsal fin dark with dark margin; anal fin pale anteriorly and slightly blackish toward tail tip. Pectoral fin mostly pale with scattered dark grayish pigment on base. Mouth cavity pale with gray peppered dots. Peritoneum pale with gray peppered dots on upper half; stomach and intestine pale.

##### Size.

The two largest specimens (998, 945 mm TL) are both ripe females with loose eggs.

##### Etymology.

The specific name is derived from the Mekong River’s estuary. In Vietnamese, the name “cửu long” means nine dragons, which is the dragon’s mouth that waters flow from to the southern sea of Viet Nam.

##### Distribution.

Only known from the type series collected in Mekong’s estuary waters, southeast coast of Viet Nam, catching by bottom trawls and scoop net (push net); they are common in the waters. The depth range is estimated to be 8–20 m.

##### Comparisons.

*Ophichthuscuulongensis* sp. nov. is similar to several of its congeners in their extremely elongated bodies. The selected characters for comparing these species are listed in Table 2. Compared to those species, *O.cuulongensis* sp. nov. has distinct vertebrae count ranging 199–207 in total. It readily differs from *O.nguyenorum* sp. nov. (190–196), *O.microcephalus* (214), and *O.rotundus* (178–184). Moreover, the new species differs from *O.congroides*, *O.chilkensis*, and *O.macrochir* in having fewer supraorbital pores (4 = 1 + 3, vs 5 = 1 + 4) and body coloration.

**Table 2.** Selected morphological and meristic characteristics of eight elongate *Ophichthus* species. Data source: 1. This study; 2. Mishra et al. 2019; 3. Kodeeswaran et al. 2023; 4. McCosker 2010; 5. Lee and Asano 1997.

	*O.cuulongensis* sp. nov.	*O.nguyenorum* sp. nov.	*O.chilkensis*	*O.congroides*	*O.macrochir*	*O.microcephalus*	*O.rutidoderma*	*O.rotundus*
**Proportions**								
HL (%TL)	5.2–6.2	5.4–6.2	4.9–5.5	8.3–8.4	5.6–6.7	4.8	5.7–7.2	5.4–6.9
Trunk (%TL)	25.3–28.5	25.9–28.0	28.1–31.3	–	25.2–31.7	31.5	26.2–27.8	30.9–31.4
Tail (%TL)	65.4–68.8	66.5–68.6	63.5–70.7	60–63	62.5–69.3	63.8	65.7–67.5	60.7–66.7
**Ratios**								
TL/BD	48.9–63.8	61.8–68.8	50.0–90.9	–	54.9–74.5	71.4	39.2–56.2	37.0–43.5
PDL/HL	1.2–1.6	1.4–1.6	1.3–1.5	1.6–1.7	1.1–1.2	1.4	1.2–1.6	–
Trunk/HL	4.2–5.4	4.3–5.0	5.5–6.1	3.5–3.8	4.4–5.5	6.5	3.7–4.7	–
Trunk/PDL	2.7–3.8	3.0–3.5	4.1–4.6	2.1–2.3	3.2–4.5	4.6	2.9–3.3	–
HL/SNL	6.5–7.8	6.1–6.8	5.4–6.1	4.9–5.1	5.1–7.3	6.9	5.9–8.0	5.8–7.2
HL/UJL	3.8–4.3	3.2–3.6	3.2–3.8	2.3	2.4–3.6	4.0	3.4–4.4	–
**Meristics**								
SO	1 + 3	1 + 3	1 + 4	1 + 4	1 + 4	–	1 + 3	1 + 3
POM	5 (rarely 6) + 2	5 or 6 + 2	5 + 2	6 + 2	4–5 + 2	–	4–6 + 2	5 + 2
PDLL	13–17	13–16	13–14*	–	11–14	–	14–17	–
PALL	62–65	61–64	69–71	78	69–71	–	59–64	65–66
Protrusion number	2 (rarely 1)	1 (rarely 2)	1	0	2	1	2 (rarely 1)	2
MVF(VF)	14-62-202	15-62-192	11-69-210	21–76–206	11–70–217	13–72–214	15–63–197	14–64–182
TV	199–207	190–196	206–214	204–208	214–221	214	195–199	178–184
**Morphology**								
Skin condition	wrinkled	wrinkled	wrinkled	smooth	wrinkled	–	wrinkled	smooth
Body coloration	brown, bicolored	dark gray	dark olive brown	dark gray	black to dark brown	–	brown, bicolored	brown
Data sources	1	1	2, 3*	1, 4	1	2	1	1, 5

*Ophichthuscuulongensis* sp. nov. is most similar to *O.rutidoderma*, both sharing a short head, relatively long tail, body depth at anus and pores on head and lateral line. However, it can be distinguished from *O.rutidoderma* by its MVF 14-62-202 (vs 15-63-197), a higher total vertebral count (199–207, vs 195–199), maxillary teeth (mostly biserial or more vs uniserial anteriorly and biserial posteriorly), and mandible teeth (biserial to triserial vs biserial anteriorly and uniserial posteriorly).

*Ophichthuscuulongensis* sp. nov. is also similar to *O.chilkensis* and *O.macrochir*, sharing a short head, a relatively long tail, an anus situated at the front of the total length, preopercuomandibular pores, and numerous longitudinal wrinkles on the body. However, it can be separated by its MVF 14-62-202 (vs 11-69-210 and 12-69-214, respectively), fewer total vertebrae (199–207, vs 206–214 and 207–221, respectively), and a shorter upper-jaw length (3.8–4.3 in HL, vs 3.2–3.8 and 2.4–3.6 in HL, respectively).

Although *Ophichthuscuulongensis* sp. nov. shares similar vertebral counts and preoperculomandibular pores with *O.congroides*, the former can be separated from the latter by having fewer lateral-line pores before anus (62–65 vs 78) and a shorter head length (5.2–6.2% TL, vs 8.3–8.4% TL) and a shorter snout length (6.5–7.8 in HL, vs 4.9–5.1 in HL) and a shorter upper-jaw length (3.8–4.3 in HL, vs 2.3 in HL) and different MVF (14-62-202 vs 21-76-206).

##### Remarks.

*Ophichthuscuulongensis* sp. nov. has some characteristics, such as tooth arrangement and the shape of protrusions, which may be caused by ontogenetic changes. The jaws are arranged in biserial and triserial rows; vomerine teeth also show variability in arrangement among materials we examined. The teeth are large and robust to fat, becoming subequal anteriorly, similar to the teeth form of *Pisodonophis* in the larger specimens. The protrusions usually number two, but the one can degenerate or become very small on the side lip.

#### ﻿ Ophichthus nguyenorum

Taxon classificationAnimaliaAnguilliformesOphichthidae

Vo, Hibino & Ho sp. nov.

63195FA1-BD9A-53E1-9D5D-56D2C09ACAAB

https://zoobank.org/9EF1D046-B202-461C-9DF7-4EDF7E149913

**Figure 3.**
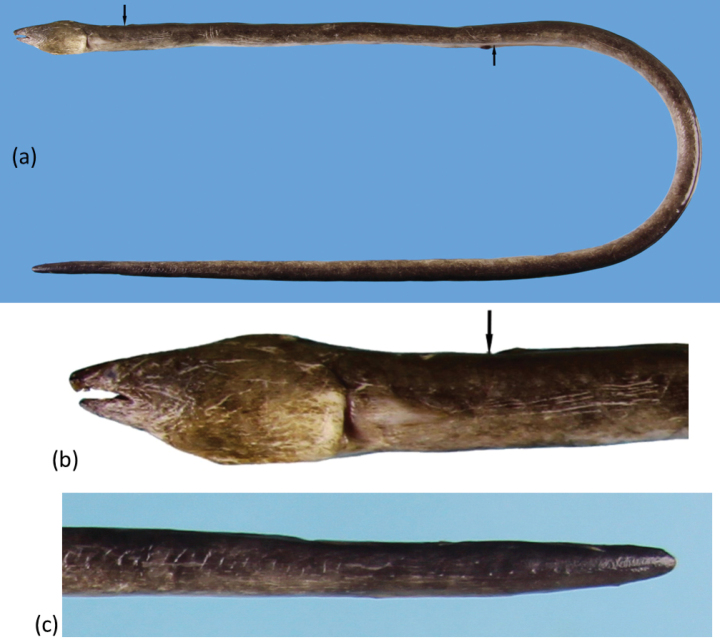
*Ophichthusnguyenorum* sp. nov. **a, b** fresh specimen of the holotype, OIM-E. 55827, 887mm TL, arrows point to the DFO and AFO, respectively **c** close-up of head view from lateral side of the paratype, OIM-E. 55827, 703 mm TL, arrow points to the DFO**d** close-up of tail view from ventral side of the holotype.

**Figure 4.**
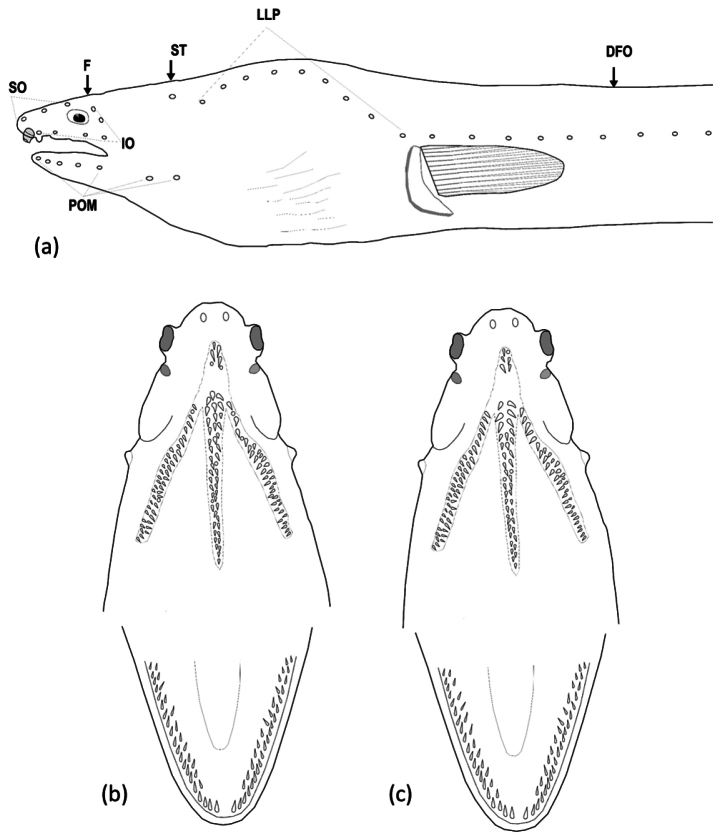
Drawing demonstrating the head pores (**a**) and teeth on both jaws (**b, c**) of *Ophichthusnguyenorum* sp. nov. **a, b** from the holotype **c** from OIM-E.55828, paratype, 708 mm TL.

Figs 3 , 4 ; Tables 1 , 2 English name: Darked Long-body Snake Eel Vietnamese name: Cá lịch dài lưng đen

##### Type material.

***Holotype*** • OIM-E.55827, 887 mm TL, field no. Q.01095-3, ca 12°19'N, 109°20'E, Đồng Hòa, Cần Giờ district, Hồ Chí Minh City, southeast coast of Vietnam, South China Sea, bottom trawl, ca 10–20 m, 10 Nov. 2023. ***Paratypes***: Seventeen specimens, 680–976 mm TL • NMMB-P41235, 5 specimens, 703–908 mm TL • KMNH VR 100623, 3 specimens, 740–848 mm TL • OIM-E.55826, 4 specimens, 784–976 mm TL, all collected with the holotype • OIM-E. 55828, 4 specimens, 697–852 mm TL • same location with holotype, bottom trawl, ca 10–20 m, 20 Jan. 2023; OIM-E. 55829, 680 mm TL • bottom trawl, ca 10–20 m, 20 Sep. 2023.

##### Diagnosis.

An extremely elongate *Ophichthus* with the following combination of characters: snout rather pointed but occipital strongly convex (duck-shaped); body with numerous longitudinal wrinkles, weak on posterior abdomen; head 5.4–6.2% TL; preanal length 31.4–33.5% TL; tail 66.5–68.6% TL; snout length 14.6–16.4% HL; one protrusion along upper lip (rarely 2 on one side); dorsal-fin origin slightly behind pectoral-fin tip; SO 1 + 3, POM 5 or 6 + 2; all teeth small and sharp; teeth on maxilla in one row anteriorly but increasing posteriorly; teeth on mandible biserial; body dark, usually including abdomen; dorsal fin darker with dark margin; anal fin initially pale but darkening towards tip; total vertebrae 190–196, MVF 15-62-192.

##### Description.

Counts and measurements of the holotype (in mm): total length 887, head 47.8, trunk 236.2, tail 601.4, predorsal length 67.4, pectoral-fin length 14.9; body depth at gill opening 14.4; body width at gill opening 13.7; body depth at anus 13.5; body width at anus 12.5; snout 7.3; upper jaw 14.1; snout overhang beyond tip of lower jaw 3.5; eye diameter 2.8; interorbital width 6.2; gill opening height 8.4; isthmus width 7.3.

An extremely elongate snake eel (Fig. 3a), subcircular to posterior portion of tail, then becoming slightly compressed, tail tip pointed and strong, its depth at gill openings 60.9 (57.6–64.2) in TL, Branchial basket slightly expanded and deeper than trunk. The skin slightly longitudinally wrinkled on body; skin folds not deep dorsally on body and weaker on posterior abdomen. Anus situated approximately in the anterior part of total length, head, and trunk 3.2 (3.0–3.2) in TL, head short 17.5 (16.1–18.6) in TL, and tail elongate 1.5 in TL. Snout short 6.4 (6.1–7.0) in HL, tip narrowly conical at dorsal view, underside of snout not bisected by a groove. Lower jaw short, its tip not extending beyond anterior margin of nostril tube; its length 4.1 (3.8–4.4) in HL. Upper jaw moderately long, rictus well behind posterior margin of eye; its length 3.4 (3.2–3.6) in HL.

Eye moderate in size, positioned above upper jaw, its diameter 4.8 (4.5–5.1) in upper jaw and 16.3 (15.1–17.3) in HL. Anterior nostril tubular, extending ventrolaterally from snout, reaching below upper lip and chin when directed downward. Posterior nostril is a hole above upper lip, covered by a broad flap that extends well below edge of upper lip. One protrusion on upper lip, positioned just behind anterior-nostril tube, rarely another one present below eye but extremely tiny and only on one side. Dorsal-fin origin behind head, by less than one pectoral-fin length behind pectoral-fin tip and 1.5 (1.4–1.6) times head length behind head. Median fins low but obvious, ending approximately one upper-jaw length before the broadly pointed tail tip. Pectoral fin wedge-shaped with a narrow base, its length less than three times its base width, broad at middle and the longest rays at mid-fin.

Head pores small but apparent (Fig. 4a). SO 1 (ethmoid) + 3 on dorsal surface of snout and interorbital space; IO 3 + 3, 1 between nostrils, two below eye and three behind eye; POM 5 or 6 + 2 (5 + 2 in holotype), the last pore slightly before rictus; F 1, ST 3. Indistinct minute sensory papillae present along nape, anterior margin of orbit, and around base of anterior nostril. Lateral-line pores apparent; HLL 8 (8–9), in an arching sequence, PDLL 15 (13–16); PALL 62 (61–64); TLL 187 (185–190), the last at approximately one jaw length in front of tail tip.

Teeth (Fig. 4b, c) small, conical, sharp. Intermaxillary with 4–6 teeth arranged in two rows (some paratypes with one row with 3 or 4 teeth) followed by 22 (18–25) teeth on vomer, with biserial rows anteriorly (some paratypes with triserial irregular rows in middle) and uniserial posteriorly, which decrease slightly in size posteriorly. Maxilla with 22 (right side) (20–24) or 23 (left side) (21–25) teeth, mostly uniserial anteriorly and becoming biserial at posterior portion with irregular rows. Mandible with 27 (right side) (24–31) or 29 (left side) (27–34) teeth arrange in one irregular row anteriorly, gradually becoming two irregular rows posteriorly, some paratypes with additional teeth anteriorly forming two irregular rows and gradually becoming one row at middle and then two irregular rows posteriorly (Fig. 4c). Predorsal vertebrae 15 (13–17), pre-anal vertebrae 63 (61–64), and total vertebrae 192 (190–196) (one paratype specimen with a broken tail, total vertebrae only 185).

Coloration. When fresh (Fig. 3a) body uniformly dark; pectoral fin pale brown with peppered dots; anal fin pale anterior and change to darker toward the ending in approximately three times head length before the tail tip. After preservation, body uniformly dark dorsally and pale brown ventrally. Dorsal fin dark with dark margin; anal fin pale white anteriorly with slightly blackish pigment toward end of tail tip. Pectoral fin mostly dark grayish with scattered pigments. Mouth cavity dark except pale tooth ridges. Peritoneum pale with gray peppered dots on upper half; stomach and intestine pale. Tail tip blackish.

##### Size.

The two largest specimens (976, 908 mm TL) are both ripe females with loose eggs.

##### Etymology.

The specific name of the new species is derived to honor three doctors with the last name Nguyen: Dr. Phung Huu Nguyen, Huong Khac Nguyen, and Thi Nhat Nguyen for their contributions to marine fish taxonomy in Viet Nam.

##### Distribution.

Only known from the type series collected from Mekong coastal region, southeast coast of Vietnam by bottom trawls. The depth range is estimated to be 10–20 m.

##### Comparisons.

*Ophichthusnguyenorum* sp. nov. is different from most congeners belonging to the species group with elongate and extremely elongate bodies. Selected characters for comparing these species are listed in Table 2. Compared to those species, it has a distinct total vertebral count range 190–196, which differs from *Ophichthuscuulongensis* sp. nov. (199–207), *O.congroides* (204–208), *O.microcephalus* (214), *O.rotundus* (178–184). Moreover, the new species differs from *O.congroides*, *O.chilkensis*, and *O.macrochir* in having fewer supraorbital pores (4 = 1 + 3, vs 5 = 1 + 4).

Although *Ophichthusnguyenorum* sp. nov. is most similar to *O.rutidoderma*, both sharing a short head, relatively long tail, body depth at anus, and pores on head and lateral line; it can be distinguished from the latter species by its body uniformly dark and less body depth (61.8–68.8 in TL, vs 39.2–56.2 in TL), count of protrusions (generally 1 vs 2) and fewer total vertebrae (190–196 vs 195–199).

*Ophichthusnguyenorum* sp. nov. is also similar to *O.chilkensis* and *O.macrochir*, sharing a short head, relatively long tail, and its anus situated at the front of total length, preopercular mandibular pores and numerous longitudinal wrinkles on the body, but it can be separated from the two species by its MVF 15-62-192 (vs 11-69-210 and 12-69-214, respectively), fewer total vertebrae (190–196, vs 206–214 and 207–221, respectively), shorter upper-jaw length (3.8–4.3 in HL, vs 3.2–3.8 and 2.4–3.6 in HL, respectively), and DFO behind the pectoral-fin tip (vs above tip of the fin). *Ophichthusnguyenorum* sp. nov. also can be separated from *O.congroides* in having fewer lateral-line pores before anus (62–64 vs 78) and a shorter head length (5.4–6.2% TL vs 8.3–8.4% TL), a shorter snout length (6.1–6.8 in HL, vs 4.9–5.1 in HL), a shorter upper-jaw length (3.2–3.6 in HL, vs 2.3 in HL), and different MVF (15-62-192 vs 21-76-206).

##### Remarks.

*Ophichthusnguyenorum* sp. nov. has some characteristics, such as body coloration, tooth arrangement, and the shape of protrusions, which may be caused by ontogenetical changes. In small sizes, the body is dark grey dorsally and pale brown ventrally; the anal fin is pale brown anteriorly in a larger paratype. The vomerine teeth are arranged in biserial rows, and the upper jaw also has biserial rows posteriorly in some specimens. The protrusions are usually two; however, the posterior one can degenerate or become very small on the side lip.

#### ﻿ Ophichthus macrochir

Taxon classificationAnimaliaAnguilliformesOphichthidae

(Bleeker, 1852)

EEA36C79-2A2B-5220-A5DE-CE5C030E9A28

**Figure 5.**
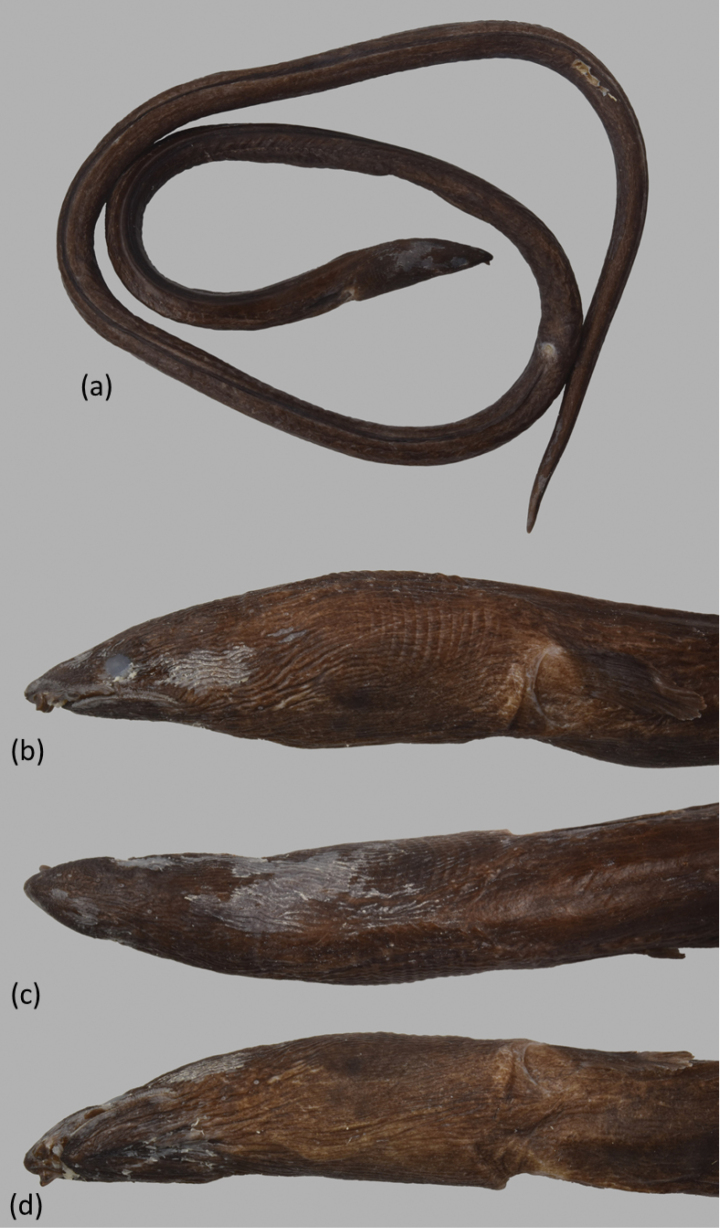
*Ophichthusmacrochir* (Bleeker, 1852), NMMB-P23577, 646 mm TL; preserved specimen **a** whole fish. Close-ups of head view from lateral side (**b**), view from dorsal (**c**) and view from ventral (**d**).

Fig. 5 , Tables 1 , 2 English name: Bigfin Snake Eel Chinese name: 大鰭蛇鰻

Ophisurus macrochir Bleeker, 1852: 26 (type locality: Jakarta, Java, Indonesia).
Ophisurus woosuitingi Chen, 1929: 22, pl. 2 (type locality: Ying Khou, Kwangtung, China).

##### Material examined.

Eight specimens, 324–824 mm TL: BMNH 1867.11.28.262 (Bleeker specimen), 501 mm TL, Jakarta, Java, Indonesia; CAS 52580, 324 mm TL, Dumaguete, Negros, Philippines • CAS 233838, 631 mm TL, Lagoon of Apulit Island, northern Palawan, Philippines • KMNH VR 100261, 659 mm TL, Dong-gang, Taiwan • NMMB-P23577, 646 mm TL, Ke-tzu-liao, Kaohsiung, Taiwan • NMMB-P24692, 356 mm TL, Dong-gang, Taiwan • NMMB-P36831, 626 mm TL, Ke-tzu-liao, Kaohsiung, Taiwan • OIM-E.55830, 544 mm TL, Kỳ Hà market (ca 15°28'20"N, 108°41'01"E), Tam Kỳ, Quảng Nam, Vietnam; OIM-E.558331, ~ 824 mm TL (tail is broken), Đồng Hòa, Cần Giờ, Hồ Chí Minh city, Vietnam.

##### Diagnosis.

An elongate *Ophichthus* with the following combination of characters: body strongly wrinkled; head length 5.0–6.7% TL; tail length 62.5–69.3% TL; two protrusions along upper lip; dorsal-fin origin at approximately same vertical through pectoral-fin tip; SO 1 + 4, POM 4–5 (usually 5) + 2; teeth on maxilla mostly uniserial, on mandible biserial; teeth on vomer bi- or triserial; body uniformly black to dark brown; dorsal and anal fins dark gray to black; total vertebrae 214–221, MVF 11-70-217.

##### Distribution.

Thailand (Gulf of Thailand), Indonesia (Java and Sumatra), Vietnam, Philippines, southern China, Taiwan, and Japan (larva only in Japan). Usually occurring in shallow water above 25 m, but specimens from Taiwan were possibly collected deeper than 100 m.

##### Remarks.

We have not examined the holotype (RMNH.PISC.7174) directly; however, McCosker (2022) noted the MVF of that holotype *O.macrochir* is 11/70/221, the only available data for the holotype. McCosker and Ho (2015) provided the range of the total number of vertebrae of the species as 207–218 in the key based on specimens collected from Taiwan. *Ophichthusmacrochir* has been recorded from several regions with morphological information (cf. Allen and Erdmann 2012; Hibino 2019; McCosker 2022), but diagnostic characters were uncertain. In addition, some elongate species have been described or redescribed as valid species in recent years (e.g., Mishra et al. 2019; this study). Based on only our examination and vertebral information of the holotype, a new diagnosis is provided herein.

*Ophisuruswoosuitingi* Chen, 1929 was originally described based on a single specimen collected from Kwangtung, southern China. We could not access the holotype of *O.woosuitingi*, but the description includes detailed information on its morphological features, with good illustrations of the head and anterior trunk. According to Chen (1929), the species has the following morphological features: strongly wrinkled body; head 6.0% TL; body depth 1.4% TL (70.8 in TL); eye 6.2% HL; snout 18.7% HL; pectoral-fin length ~ 20% HL; dorsal-fin origin slightly behind the tip of the pectoral fin; SO ?1 (ethmoid not obvious) + 4, POM 5 + 2; preanal lateral-line pores 68; teeth on maxilla mostly uniserial, on mandible biserial; teeth on vomer bi- or triserial; body color uniformly blackish. It is evident that *O.woosuitingi* is a junior synonym of *O.macrochir*, although Tang and Zhang (2004) considered this name to be valid and separated it from *O.macrochir*.

Based on our extensive examination of *O.macrochir*, including specimens from various localities in the northwestern Pacific, we were unable to differentiate it from the Indian species *O.chilkensis* based on the counts and measurements except for the protrusions on the upper lip (1 vs 2; Table 2). According to the redescription by Mishra et al. (2019), differences in fin coloration can distinguish *O.chilkensis* from *O.macrochir* (dark gray to black vs dull white with posterior 1/3 of anal fin dark). Our investigation shows that the distribution of *O.macrochir* is restricted to the western Pacific, and the Indian records of *O.macrochir* might have been misidentifications of *O.chilkensis*.

#### ﻿ Ophichthus rutidoderma

Taxon classificationAnimaliaAnguilliformesOphichthidae

(Bleeker, 1852)

0981B0CE-48F5-571B-AC9C-95423FDD1580

**Figure 6.**
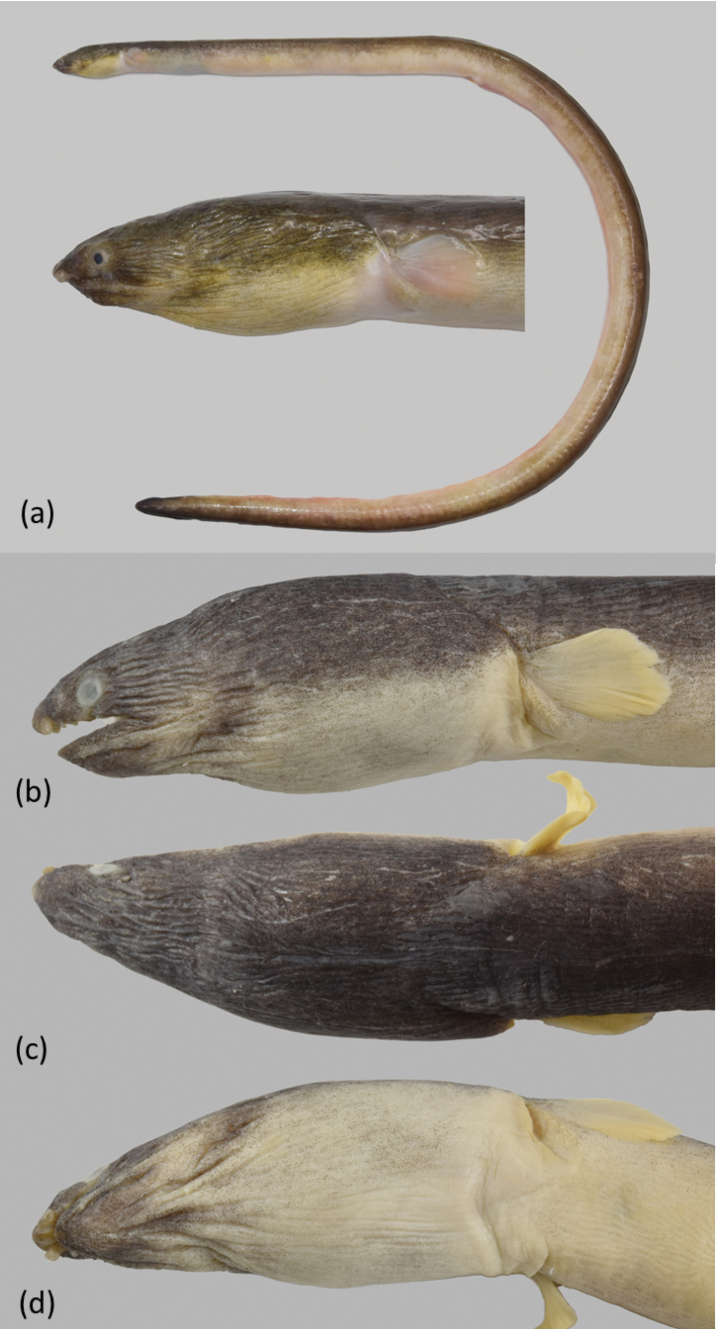
*Ophichthusrutidoderma* (Bleeker, 1852), KMNH VR 100624, 748 mm TL**a** fresh specimen, whole fish and close-up of head. Close-ups of head view from lateral side (**b**), view from dorsal (**c**), and view from ventral (**d**).

Fig. 6 , Tables 1 , 2 English name: Olive Snake Eel

Ophisurus rutidoderma Bleeker, 1852: 30 (type locality: Jakarta, Java, Indonesia); Günther 1870:63 (as Ophichthysrhytidoderma, unjustified emendation).
Ophisurus rutidodermatoides Bleeker, 1852: 31 (type locality: Jakarta, Java, Indonesia); Günther 1870:62 (as Ophichthysrhytidodermatoides, unjustified emendation).
Ophisurus lumbricoides Bleeker, 1852: 32 (type locality unknown).
Ophisurus macclellandi Bleeker, 1852: 33 (type locality: Jakarta, Java, Indonesia).

##### Type material.

***Holotype*** • BMNH 1867.11.28.226, 945 mm TL, Jakarta, Java, Indonesia (only vertebral examination).

##### Other material examined.

10 specimens, 415–867 mm TL: BMNH 1867.11.28.278, 569 mm TL, Jakarta, Java, Indonesia (Bleeker specimen of *O.maclellandi*) • BMNH 1867.11.28.292, 610 mm TL, Jakarta, Java, Indonesia (holotype of *O.rutidodermatoides*; only vertebral examination) • BMNH 1867.11.28.300, 415 mm TL (holotype of *Ophisuruslumbricoides*; only vertebral examination), Jakarta, Java, Indonesia • KAUM–I. 39446, 867 mm TL, off Tanjung Sepat, Selangor, Malaysia • KMNH VR 100624–627, 4 specimens, 569–748 mm TL, purchased at Bagan Datuk, collected off mouth of Perak River, Perak, Malaysia • OIM-E.55787, 278 mm TL, Cần Giờ fishing grounded, Hồ Chí Minh city, 15–17 m, Vietnam • OIM-E.55832, 590 mm TL, Bình Đại food market (ca 10°11'15"N, 106°41'40"E), Bình Đại District, Bến Tre province, Vietnam • OIM-E.55833, 700 mm TL, Đồng Hòa, Cần Giờ District, Hồ Chí Minh City, Vietnam.

##### Diagnosis.

An elongate *Ophichthus* with the following combination of characters: body with numerous longitudinal wrinkles, more than five longitudinal wrinkles on posterior part of eye; head length 5.7–7.2% TL; tail length 65.7–67.5% TL; two protrusions along upper lip (rarely 1 on one side); dorsal-fin origin behind pectoral-fin tip by less than one pectoral-fin length; SO 1 + 3; POM 4–6 (usually 5) + 2; teeth on maxilla uniserial initially, becoming biserial posteriorly; vomerine and dentary teeth biserial anteriorly, uniserial posteriorly; body bicolored; dorsal fin dark with narrow margin entirely, anal fin pale except ending; total vertebrae 195–199, MVF 15-63-197 (*n* = 9).

##### Distribution.

Southern Vietnam, Java, Indonesia, and Malay Peninsula of Malaysia. It is a shallow water species, collected adjacent to a river mouth. Collecting depth is estimated to be 5–20 m in Vietnam and Malaysia.

##### Remarks.

*Ophichthusrutidoderma* has been recorded only from the South China Sea and western Australia; however, there are no detailed descriptions (usually only the name in list) except the original descriptions including several synonyms and a young specimen record by Vo et al. (2019) from Ho Chi Minh City, southern Vietnam. In Vo et al. (2019), the young Vietnamese specimen (278 mm TL) was reported as the first record from Vietnam, accompanied by a detailed morphological description. Although they described the specimen as having SO 1 + 4 and maxillary teeth arranged in a single row, the correct characteristics are SO 1 + 3 and a maxilla with primarily one row of teeth, except at the posterior end, whereas an additional row of four teeth is present. McCosker (2007) noted the vertebral information of several types, but he did not mention other characters. The record from Western Australia is based on the original description of *Ophichthusderbyensis* (holotype: 258 mm TL) by Whitley (1941). While its VF closely matches that of *O.rutidoderma*, the locality of *O.derbyensis* is significantly distant from the South China Sea. Moreover, *O.derbyensis* differs in dental morphology, with a completely single row of vomerine teeth (vs 2 anteriorly and 1 posteriorly in *O.rutidoderma*), and maxillary teeth that are biserial in the anterior-middle region but uniserial posteriorly (vs uniserial anteriorly and biserial posteriorly in *O.rutidoderma*). As such, we tentatively exclude *O.derbyensis* from synonymy with *O.rutidoderma*. Actually, *O.rutidoderma* appears to be a unique species restricted to the southwestern part of the South China Sea extending to the western coast of the Malay Peninsula. Future studies to determine the validity of *O.derbyensis* are required.

## Supplementary Material

XML Treatment for Ophichthus cuulongensis

XML Treatment for Ophichthus nguyenorum

XML Treatment for Ophichthus macrochir

XML Treatment for Ophichthus rutidoderma

## ﻿Additional information

### Author ORCIDs

Quang Van Vo https://orcid.org/0000-0002-9680-6898

Yusuke Hibino https://orcid.org/0000-0002-5670-3851

Hsuan-Ching Ho https://orcid.org/0000-0003-1154-601X

Thao Thu Thi Le https://orcid.org/0000-0002-9985-4668

Ying Giat Seah https://orcid.org/0000-0002-2976-4448

### Data availability

All of the data that support the findings of this study are available in the main text.
